# Phosphatidylinositol transfer protein-α in platelets is inconsequential for thrombosis yet is utilized for tumor metastasis

**DOI:** 10.1038/s41467-017-01181-4

**Published:** 2017-10-31

**Authors:** Liang Zhao, Chelsea L. Thorsheim, Aae Suzuki, Timothy J. Stalker, Sang H. Min, Lurong Lian, Gregory D. Fairn, Shamshad Cockcroft, Amy Durham, Sriram Krishnaswamy, Charles S. Abrams

**Affiliations:** 1 Department of Medicine, School of Medicine, University of Pennsylvania, Philadelphia, PA 19104 USA; 2 St. Michael’s Hospital, Toronto, ON Canada M5B 1W8; 3 Division of Bioscience, University College London, London, WC1E 6BT UK; 4 School of Veterinary Medicine, University of Pennsylvania, Philadelphia, PA 19104 USA; 5 Children’s Hospital of Philadelphia, Philadelphia, PA 19104 USA; 6 Department of Pathology, School of Medicine, University of Pennsylvania, Philadelphia, PA 19104 USA

## Abstract

Platelets are increasingly recognized for their contributions to tumor metastasis. Here, we show that the phosphoinositide signaling modulated by phosphatidylinositol transfer protein type α (PITPα), a protein which shuttles phosphatidylinositol between organelles, is essential for platelet-mediated tumor metastasis. PITPα-deficient platelets have reduced intracellular pools of phosphoinositides and an 80% reduction in IP_3_ generation upon platelet activation. Unexpectedly, mice lacking platelet PITPα form thrombi normally at sites of intravascular injuries. However, following intravenous injection of tumor cells, mice lacking PITPα develop fewer lung metastases due to a reduction of fibrin formation surrounding the tumor cells, rendering the metastases susceptible to mucosal immunity. These findings demonstrate that platelet PITPα-mediated phosphoinositide signaling is inconsequential for in vivo hemostasis, yet is critical for in vivo dissemination. Moreover, this demonstrates that signaling pathways within platelets may be segregated into pathways that are essential for thrombosis formation and pathways that are important for non-hemostatic functions.

## Abstract (web summary)

Platelets support tumor metastasis formation. Here, the authors show that the phosphoinositide signaling modulated by PITPα is essential for platelet-mediated tumor metastasis signaling, but surprisingly it is dispensable in hemostasis in mice.

## Introduction

Platelets are best known for their contribution to hemostasis, but several lines of evidence indicate that they also contribute to tumor metastasis. For example, elevated platelet counts are associated with a poor prognosis in patients with cancer, whereas low platelet counts are associated with reduced metastatic burden^1–6^. Additionally, studies suggest that platelets assist tumor cell adhesion to the vasculature and enhance tumor cell growth. Aberrant platelet activation and aggregation are frequently found in the vasculature of cancer patients, especially in patients with metastatic tumors^7–10^. Furthermore, studies that use genetically engineered mice with altered platelet functions, or those that use pharmacologic inhibitors against platelet activation, show that platelets can protect circulating tumor cells from elimination by the immune system and therefore support further metastasis^11, 12^.

Platelet-mediated vascular plug formation is critically dependent on phosphatidylinositol signaling, although it only comprises about 8% of the phospholipids contained in a typical mammalian cell^13–17^. The phosphatidylinositol head group is capable of being differentially phosphorylated, generating a family of seven functionally distinct phospholipids, called phosphoinositides (PtdIns)^13^. Intracellular PtdIns concentrations are tightly regulated both in time and space, and the distribution between the various PtdIns species changes within seconds after agonist stimulation. PtdIns generation is a key cellular event in all eukaryotic cells, including in platelets, where it regulates integrin activation, actin assembly, and secretion^13, 14^. However, whether platelet PtdIns signaling contributes to platelet-mediated tumor metastasis is unknown.

Since PtdIns contain hydrophobic acyl side chains that render them insoluble in the aqueous cytoplasm, they are assumed to always be associated within a lipid bilayer. Curiously, the sites of synthesis of different PtdIns are on the surfaces of distinct cellular organelles. For example, phosphatidylinositol is synthesized in the endoplasmic reticulum (ER), while PtdIns(4)P is synthesized on the surface of internal granules, such as on endosomes, and PtdIns(4,5)P_2_ is synthesized on the cell membrane^18, 19^. This implies that the sequential synthesis of polyphosphorylated phosphatidylinositols, such as PtdIns(4,5)P_2_, begins with phosphatidylinositol production in the ER, and the intermediate isoforms are trafficked between the organelles before final production in the plasma membrane. We and others have shown that the sequential enzymatic steps required for higher-order phosphoinositide synthesis occur within seconds^14, 20, 21^. Consequently, a mechanism for rapidly transferring phosphatidylinositol between membranes is required.

Phosphatidylinositol transfer proteins (PITP) facilitate the transfer of insoluble phosphatidylinositol from one membrane to another in vitro. They achieve this by binding and encompassing the fatty acid side chains of phospholipids and transporting the phospholipids throughout the cytoplasm. Mammalian cells contain both PITPα and PITPβ isoforms, which are small, highly conserved, and ubiquitously expressed single PITP domain soluble proteins. Ablation or knock down of PITPα impairs viability, membrane trafficking, and cytokinesis^22–25^. Because the phosphatidylinositol transfer activity of PITPα is required for the synthesis of phosphoinositides and for the regulation of PtdIns kinases, it has been assumed that PITPα is essential for all of the signaling events that utilize phosphoinositides, including platelets^26, 27^.

In this study, we investigate whether platelet-specific disruption of phosphatidylinositol metabolism mediated by PITPα impacts hemostatic and non-hemostatic functions of platelets in tumor dissemination. Our study unexpectedly shows that PITPα, the major PITP isoform in murine platelets, is inconsequential for hemostasis in vivo. However, PITPα-mediated PtdIns(4,5)P_2_ synthesis and IP_3_ production in platelets are critical to promote platelet-mediated lung metastasis of intravenous injected tumor cells. We demonstrate that signaling pathways required for thrombosis formation may be independent from those pathways required for non-hemostatic platelet functions, such as those pathways that regulate interactions with immune cells.

## Results

### Conditional deletion of the PITPα in murine platelets

**Fig. 1:**
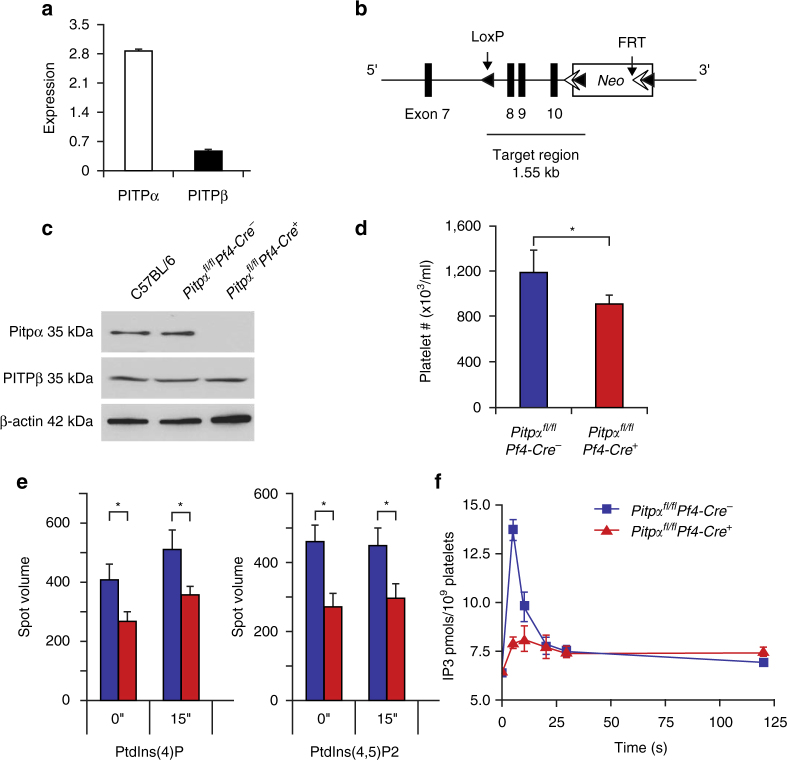
PITPα mediates PtdIns synthesis and IP_3_ production in mouse platelets. **a** Quantification of immunoblot demonstrates the types of class I PITP expressed in platelet lysates using isoform-specific antibodies. Shown is the relative abundance of individual PITP isoforms in platelets from wild-type mice when normalized to recombinant PITP protein standards (*n* = 3 mice per group; error bars are standard deviation (s.d.)). **b** Schematic representation of the conditional targeting strategy. Exons 8, 9, and 10 were targeted by the insertion of loxP recombination sites to generate *Pitpα* floxed alleles (*Pitpα* ^*fl/fl*^). **c** Immunoblot of platelet lysate demonstrating the specific deletion of the PITPα isoform in *Pitpα* ^*fl/fl*^ *Pf4-Cre* ^*+*^ platelets. **d** CBC analyses show decreased platelet count in *Pitpα* ^*fl/fl*^ *Pf4-Cre* ^*+*^ mice compared to their littermate controls (*n* = 3 mice per group; error bars are s.d.; *p < 0.05, unpaired Student’s *t*-test). **e** PtdIns synthesis in thrombin-stimulated platelets derived from *Pitpα* ^*fl/fl*^ *Pf4-* *Cre* ^−^ and *Pitpα* ^*fl/fl*^ *Pf4-Cre* ^*+*^ mice. Pooled analysis of experiments shows the effect of the loss of PITPα on PtdIns(4)P and PtdIns(4,5)P_2_ generation following thrombin stimulation at 1 U/ml (*n* = 3 mice per group; error bars are s.d.; *p < 0.05, unpaired Student’s *t*-test). **f** The effect on IP_3_ production by the loss of PITPα was analyzed. IP_3_ production was impaired even at the earliest analyzed time point (*n* = 3 mice per group; error bars are s.d.)

Platelets, like other mammalian cells, contain both the PITPα and PITPβ isoforms. We observed that the PITPα isoform is approximately six-fold more abundant than PITPβ in murine platelets (Fig. 1a and Supplementary Fig. 1). To determine the role of PITPα in platelets, we genetically deleted PITPα from murine platelets and megakaryocytes (Fig. 1b). To accomplish this, the PITPα gene was conditionally targeted at the domain previously demonstrated to be essential for the phosphatidylinositol transfer function of PITPα^22, 28^, and the resultant mice were crossed with transgenic mice expressing the CRE recombinase, whose expression was controlled by the megakaryocyte-specific platelet-factor-4 promoter (*Pf4-Cre*)^29^. We found no detectable full-length or truncated forms of PITPα protein in the lysates of platelets derived from Pf4-Cre-positive PITPα floxed mice (*Pitpα* ^*fl/fl*^ *Pf4-Cre* ^*+*^) by immunoblotting. The PITPβ protein expression remained unchanged in the *Pitpα* ^*fl/fl*^ *Pf4-Cre* ^*+*^ mice, with similar expression levels to those in the control *Pitpα* ^*fl/fl*^ *Pf4-* *Cre* ^−^ mice (Fig. 1c).

The *Pitpα* ^*fl/fl*^ *Pf4-Cre* ^*+*^ mice were grossly normal, with body weight, organ morphology, leukocyte counts, and hemoglobin levels that were indistinguishable from the littermate controls (*Pitpα* ^*fl/fl*^ *Pf4-Cre* ^−^ mice) (Supplementary Fig. 2). Although there was a 15% decrease in platelet counts in the *Pitpα* ^*fl/fl*^ *Pf4-Cre* ^*+*^ mice (Fig. 1d), it is still within normal range. The mice grew to adulthood without any observable increase in spontaneous hemorrhage, thrombosis, or mortality.

### PITPα is essential for platelet phosphoinositide signaling

To study the role of PITPα in the synthesis of phosphoinositides in platelets, we analyzed the concentration of specific phosphoinositides within platelets from *Pitpα* ^*fl/fl*^ *Pf4-Cre* ^*+*^ mice by using thin-layer chromatography (TLC). These platelets had a 34.7% reduction of PtdIns(4)P and a 41.2% reduction in PtdIns(4,5)P_2_ at resting conditions (Fig. 1e). When stimulated with thrombin, the deficiency remained in PITPα-null platelets (30.5% reduction of PtdIns(4)P levels and 34.1% reduction of PtdIns(4,5)P_2_ levels), demonstrating that PITPα is required for the synthesis of higher-order phosphoinositides in platelets.

Following thrombin stimulation, PtdIns(4,5)P_2_ in platelets is hydrolyzed by phospholipase C to generate second messengers, such as IP_3_. We analyzed IP_3_ synthesis in thrombin-stimulated platelets that lack PITPα, in order to test whether phosphoinositide transfer is required for rapid IP_3_ production. Although the baseline IP_3_ level in platelets derived from *Pitpα* ^*fl/fl*^ *Pf4-Cre* ^*+*^ mice was not different from the control mice, the IP_3_ generation following thrombin stimulation was blunted by approximately 80%, an effect that was apparent as early as 5 s after agonist stimulation (Fig. 1f). Furthermore, the intracellular calcium levels were lower in *Pitpα* ^*fl/fl*^ *Pf4-Cre* ^*+*^ platelets following thrombin stimulation (Supplementary Fig. 3). These data indicate that PITPα-mediated synthesis of PtdIns(4,5)P_2_ contributes to IP_3_ formation, which occurs within seconds after agonist stimulation. It is noteworthy that although PITPα is only required for the synthesis of approximately 40% of platelet PtdIns(4,5)P_2_ (Fig. 1e), it is required for a much larger percentage of the IP_3_ that is synthesized after agonist stimulation (Fig. 1f). Since IP_3_ is derived entirely from PtdIns(4,5)P_2_, these results suggest that PITPα may be required for the production of the specific pool of PtdIns(4,5)P_2_ that is utilized for IP_3_ formation.

### PITPα is inconsequential for platelet aggregation

**Fig. 2:**
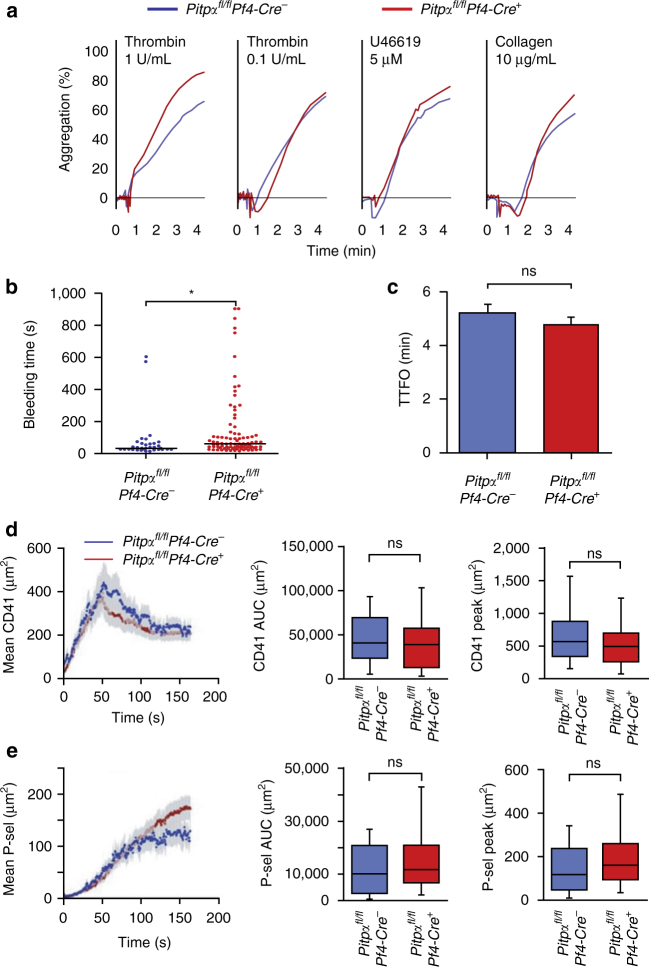
Platelet PITPα is inconsequential for hemostasis. **a** Platelets lacking PITPα were analyzed after agonist stimulation in a Lumi-Dual aggregometer. Platelets derived from *Pitpα* knockout mice exhibited essentially no defect in aggregation in response to all analyzed agonists. **b** Tail bleeding times in mice lacking PITPα. *Pitpα* ^*fl/fl*^ *Pf4-Cre* ^*+*^ mice (*n* = 90) and *Pitpα* ^*fl/fl*^ *Pf4-* *Cre* ^−^ mice (*n* = 40) were analyzed for the duration of their tail bleeding. The median difference was 29.5 s (*p < 0.005, unpaired Student’s *t*-test). **c** Carotid arteries of *Pitpα* ^*fl/fl*^ *Pf4-Cre* ^*+*^ mice (*n* = 5) and *Pitpα* ^*fl/fl*^ *Pf4-* *Cre* ^−^ mice (*n* = 7) were subjected to a ferric chloride induced injury, and blood flow was monitored as a measure of thrombus formation. The time-to-form occlusions (TTFO) of the blood flow were not found to be significantly different between mice lacking PITPα in their platelets and the control mice. Statistical analysis was performed using an unpaired Student’s *t*-test. Error bars are s.d. (**d**, **e**). Laser-induced injury model demonstrates normal in vivo thrombosis and platelet secretion in *Pitpα* ^*fl/fl*^ *Pf4-Cre* ^*+*^ mice. Shown is platelet accumulation (**d**) and P-selectin exposure (**e**) in response to a laser-induced injury to mouse cremaster arterioles. The area under the area/time curve and peak area are shown. The graphs show the means for *n* = 15 injuries in three *Pitpα* ^*fl/fl*^ *Pf4-* *Cre* ^−^ mice and *n* = 25 injuries in three *Pitpα* ^*fl/fl*^ *Pf4-Cre* ^*+*^ mice. Statistical analysis was performed using an unpaired Student’s *t*-test. Shown are the means ± s.d. for all figure panels

To determine whether the deletion of PITPα impacts platelet function, we compared the ex vivo aggregation of PITPα-null platelets with those of the littermate controls using light transmission aggregometry. PITPα-null platelets aggregated normally in response to the platelet agonists that include thrombin, collagen, ADP, PMA, and U46619, at all dosages tested (Fig. 2a and Supplementary Table 1). Ex vivo platelet αIIbβ3 integrin activation (JON/A) and secretion (P-selectin) measured by flow cytometry were also normal (Supplementary Table 2). These results were unexpected given the defects in phosphoinositide synthesis and second messenger formation observed in PITPα-null platelets.

To determine whether these results also pertained to platelet function in vivo, we investigated whether platelets lacking PITPα formed thrombi normally by using three established murine models. First, we analyzed whether the loss of PITPα in platelets affected tail bleeding times. By analyzing a large number of animals (90 *Pitpα* ^*fl/fl*^ *Pf4-Cre* ^*+*^ mice and 40 *Pitpα* ^*fl/fl*^ *Pf4-Cre* ^−^ mice), we could demonstrate that the bleeding time was longer in the *Pitpα* ^*fl/fl*^ *Pf4-Cre* ^*+*^ mice (median time 58.5 s) when compared with the *Pitpα* ^*fl/fl*^ *Pf4-*Cre^−^ mice (median time = 29.0 s, *p* < 0.005, unpaired Student’s *t*-test; Fig. 2b). However, the average blood volume lost was comparable between the *Pitpα* ^*fl/fl*^ *Pf4-Cre* ^*+*^ and *Pitpα* ^*fl/fl*^ *Pf4-* *Cre* ^−^ mice (Supplementary Fig. 4). Since the absolute differences in bleeding time were small, there was a large amount of variability in this assay even within mice of the same genotype, and the results may be influenced by platelet-independent factors such as coagulation and vascular muscle tone, we analyzed the ability of these mice to form intravascular thrombi using several alternative methods.

Platelet-mediated in vivo thrombosis were directly tested using a FeCl_3_ carotid injury model by analyzing thrombus formation in response to a chemical injury. Using this method, we could not identify any difference in the rate of thrombus formation between *Pitpα* ^*fl/fl*^ *Pf4-Cre* ^*+*^ mice and *Pitpα* ^*fl/fl*^ *Pf4-* *Cre* ^−^ mice (Fig. 2c). We also analyzed thrombosis formation and platelet α-granule secretion in response to a laser-induced vascular injury^30^. As shown in Fig. 2d, the loss of platelet PITPα did not significantly decrease platelet accumulation at the injured vessel wall. Similarly, PITPα-null platelets translocated normal amounts of P-selectin onto their surface (Fig. 2e), indicating that these genetically modified platelets have no obvious defect in α-granule secretion in vivo, confirming the previous ex vivo secretion data. Taken together, our studies demonstrate that the predominant platelet PITP isoform, PITPα, does not contribute significantly to platelet-mediated hemostasis ex vivo or in vivo.

### Platelet PITPα is essential for melanoma cell metastasis

**Fig. 3:**
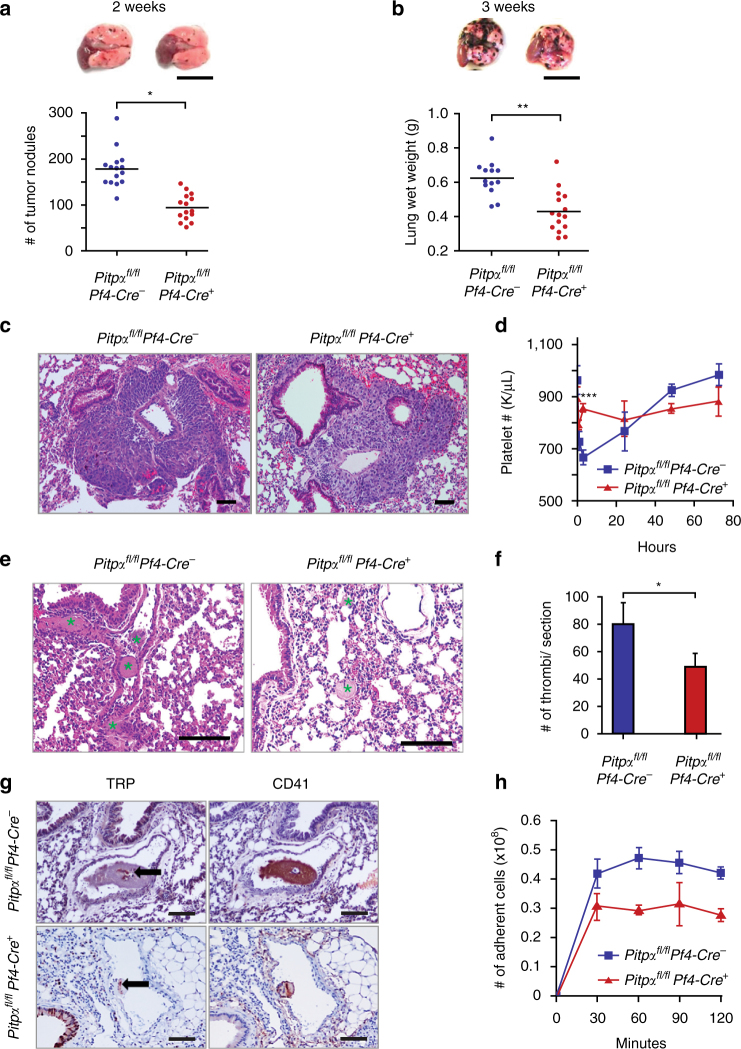
Loss of PITPα in platelets impairs tumor metastasis formation. **a** The lungs derived from *Pitpα* ^*fl/fl*^ *Pf4-Cre* ^−^ and *Pitpα* ^*fl/fl*^ *Pf4-Cre* ^*+*^ mice harvested 2 weeks after tail vein injection with B16F10 melanoma cells. Shown are representative lungs. The number of tumor nodules on the lung surface was reduced in *Pitpα* ^*fl/fl*^ *Pf4-Cre* ^*+*^ mice when compared with *Pitpα* ^*fl/fl*^ *Pf4-Cre* ^−^ controls. Scale bar is 10 mm. *p < 0.0001, unpaired Student’s *t*-test. **b** Similarly, 3 weeks after tumor injection, the lungs of the *Pitpα* ^*fl/fl*^ *Pf4-Cre* ^*+*^ mice contained less metastasis than the *Pitpα* ^*fl/fl*^ *Pf4-* *Cre* ^−^ mice as indicated by their weights. Scale bar is 10 mm. **p < 0.0001, unpaired Student’s *t*-test. **c** H&E staining of tumors in lung tissue sections at 3 weeks after tumor injection show no significant fibrosis or hemorrhage. Black scale bars represent 100 µm. **d** Shown are serial platelet counts analyzed after tumor injection. Control mice have an abrupt decrease in their platelet counts 3 h after tumor injection. This reduction was markedly blunted in the *Pitpα* ^*fl/fl*^ *Pf4-Cre* ^*+*^ mice (*n* = 6 for each genotype, ***p < 0.05, unpaired Student’s *t*-test). Shown are the means ± s.d. **e** H&E staining for lung tissues at 3 h after tumor cell injection demonstrated tumor-induced thrombi formation within lung tissue. The stars indicate the tumor-induced thrombi. Black scale bars represent 200 µm. **f** Quantification of CD41-positive thrombi formation observed within lung tissue of the *Pitpα* ^*fl/fl*^ *Pf4-Cre* ^*+*^ mice (*n* = 4) and the *Pitpα* ^*fl/fl*^ *Pf4-Cre* ^−^ control mice (*n* = 5). ***p = 0.02, unpaired Student’s *t*-test. Error bars are s.d. **g** Immunohistochemistry analysis of tumor-induced thrombi in lung tissues at 3 h after tumor injection staining for CD41 (platelet marker) and TRP1 (B16F10 tumor cells). The arrows indicate the tumor cells. This demonstrates that the tumor cells are coated with large clusters of platelets in the *Pitpα* ^*fl/fl*^ *Pf4-* *Cre* ^−^ mice. This phenomenon was less frequently found in the *Pitpα* ^*fl/fl*^ *Pf4-Cre* ^*+*^ mice. Black scale bars represent 100 μm. **h** Ex vivo adhesions of platelets on tumor cell layers indicate impaired interactions between *Pitpα* ^*fl/fl*^ *Pf4-Cre* ^*+*^ platelets and tumor cells. *N* = 3 mice per group. Shown are the mean ± s.d. for all figure panels

In addition to hemostasis, platelets have been implicated in other processes, such as the formation of tumor metastases^31^. To determine whether PITPα-mediated phosphoinositide metabolism in platelets is involved in tumor dissemination, we utilized a well-characterized B16F10 melanoma model of tumor metastasis^32^. In this model, the quantity of tumor foci were measured following intravenous injection of B16F10 melanoma cells in mice. Two weeks after tumor cell injection, *Pitpα* ^*fl/fl*^ *Pf4-Cre* ^*+*^ mice developed 50% fewer tumor foci on the surface of their lungs as compared to the littermate controls (*p* < 0.0001, unpaired Student’s *t*-test; Fig. 3a). Furthermore, 3 weeks after tumor cell injection, the lung weights of freshly dissected *Pitpα* ^*fl/fl*^ *Pf4-Cre* ^*+*^ mice were 50% lower than those harvested from *Pitpα* ^*fl/fl*^ *Pf4-* *Cre* ^−^ mice (*p* < 0.0001, unpaired Student’s *t*-test; Fig. 3b). The histology showed that compared to the *Pitpα* ^*fl/fl*^ *Pf4-Cre* ^*+*^ mice, *Pitpα* ^*fl/fl*^ *Pf4-* *Cre* ^−^ mice did not develop more hemorrhages or fibrosis in their lungs at 3 weeks after tumor injection (Fig. 3c). This suggested that the increased lung weight in the control mice is due to the metastatic burden of the tumors. Thus, these data indicate that platelet PITPα contributes to the dissemination of intravenous injected B16F10 melanoma cells.

To determine whether this observation was limited to B16F10 melanoma cells, Lewis lung carcinoma (LLC) cells also were injected intravenously, and metastasis formation was evaluated at 2 weeks after tumor injection by counting the number of tumor foci on the lung surface (Supplementary Fig. 5). Again, *Pitpα* ^*fl/fl*^ *Pf4-Cre* ^*+*^ mice developed significantly less lung metastasis when compared to their littermate controls, demonstrating that the relationship between *Pitpα* ^*fl/fl*^ *Pf4-Cre* ^*+*^ platelets and metastasis formation is not unique to a specific tumor cell line.

One possible mechanism by which platelet PITPα contributes to the dissemination of tumor cells was revealed as control mice, but not *Pitpα* ^*fl/fl*^ *Pf4-Cre* ^*+*^ mice, quickly become thrombocytopenic just 3 h after injecting the tumor cells (Fig. 3d). Platelet counts in the control mice dropped 70%, while *Pitpα* ^*fl/fl*^ *Pf4-Cre* ^*+*^ mice had relatively stable platelet counts. Histologic analyses of the lung tissue at the 3 h time point after tumor cell injection revealed that *Pitpα* ^*fl/fl*^ *Pf4-Cre* ^*+*^ mice have significantly fewer (Fig. 3e, f) and smaller (Fig. 3g) thrombi in their lungs and pulmonary vasculature than those found in the control mice. These data suggest that the formation of thrombi mediated by platelet PITPα contributes to the thrombocytopenia induced by the tumor cells.

Previous studies have supported the hypothesis that a direct in vivo interaction (adhesion) between platelets and tumor cells can facilitate the implantation of the metastasis and promote tumor cell invasion and propagation^33, 34^. To determine whether PITPα facilitates platelet interactions with B16F10 tumor cells, we characterized the pulmonary thrombi in more detail by immunohistochemical staining at 3 h after tumor injection. We observed that at the core of these thrombi, there were a small number of tumor cells that were enveloped by platelets. This shroud of platelet-rich thrombi was much larger in the control mice than that found in the *Pitpα* ^*fl/fl*^ *Pf4-Cre* ^*+*^ mice (Fig. 3g). Correspondingly, PITPα-null platelets have significantly reduced ex vivo adhesion to tissue-cultured tumor cells than PITPα wild-type platelets (Fig. 3h). Together, these findings suggest that PITPα in platelets promotes platelet–tumor interaction and platelet-rich thrombi around tumors.

### Tumor-induced thrombin and fibrin generation requires PITPα

**Fig. 4:**
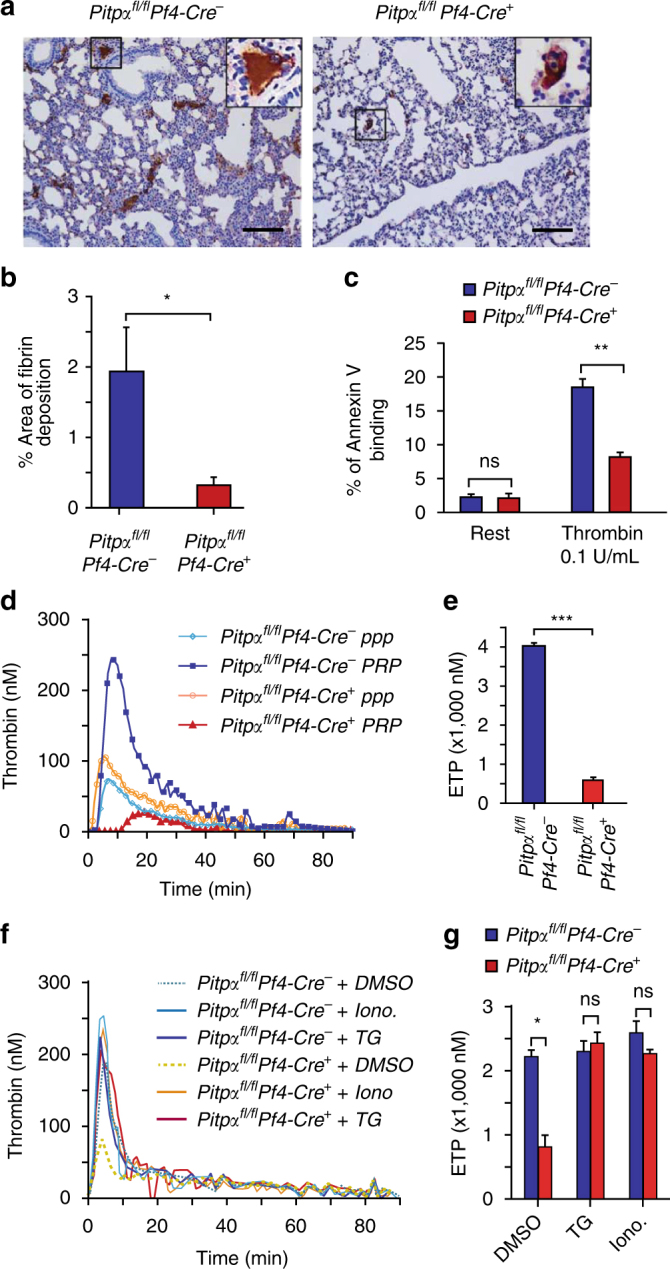
Loss of PITPα in platelets impairs fibrin deposition and thrombin generation. **a** Immunohistochemistry staining for fibrin in tumor-induced thrombi found in lung tissue at 3 h after tumor injection. The brown color indicates fibrin deposition within the tissue. Black scale bars represent 100 µm. **b** Quantification of fibrin deposition based on immunohistochemistry staining. *N* = 3 mice per group. *p < 0.05, unpaired Student’s *t*-test. Error bars are s.d. **c** Platelets lacking PITPα have an impaired ability to bind Annexin V ex vivo. The mean values are averaged from four independent experiments. **p = 0.001, unpaired Student’s *t*-test. Error bars are s.d. **d** Ex vivo TGA initiated by B16F10 tumor cells. Shown is representative kinetics of thrombin generation in PRP and in PPP. **e** The endogenous thrombin potential (ETP) shown is the mean value of total thrombin generated in PRP, containing either PITPα wild-type platelets (blue bar) or PITPα-null platelets (red bar). *N* = 3 mice per group. ***p = 0.01, unpaired Student’s *t*-test. Error bars are s.d. **f**, **g** Thrombin generation initiated by B16F10 tumor cells in the presence of agonist TG and calcium ionophore (Iono.). *N* = 3 mice per group. *p < 0.05, unpaired Student’s *t*-test. Error bars are s.d

Since hemostatic plugs are often composed of aggregated platelets that are woven together by fibrin, we analyzed the tumor-induced thrombi for fibrin by immunohistochemistry. We observed that the area of fibrin staining was about six-fold higher in the lung tissues of the control mice when compared to the *Pitpα* ^*fl/fl*^ *Pf4-Cre* ^*+*^ mice (Fig. 4a, b).

Platelets support fibrin formation by providing a negatively charged surface for the prothrombinase components (FVa and FXa) to assemble and activate thrombin. This occurs once platelets flip the negatively charged phospholipid phosphatidylserine from the inner leaflet of their cell membrane to the outer leaflet. Using the Annexin V-binding assay to detect phosphatidylserine exposure on the outer membrane leaflet of platelets, we observed that Annexin V-binding was significantly reduced in PITPα-null platelets after activation by agonists (thrombin at 0.1 U/ml and collagen at 5 µg/ml) when compared to the control platelets (Fig. 4c). This indicates that PITPα*-*null platelets have an impaired ability to externalize negatively charged lipids on their surface, which in turn cause defects in prothrombinase complex assembly, prothrombin activation, and ultimately fibrin generation.

B16F10 melanoma cells have been shown to express high levels of tissue factor on their surface, thereby allowing these tumor cells to initiate the coagulation cascade^35^. To analyze the contribution of platelet PITPα to B16F10 melanoma cell-induced prothrombin activation, we utilized a well-described fluorometric thrombin generation assay (TGA) to investigate the impact of platelet PITPα on thrombin generation in platelet-rich plasma (PRP). When tumor cells were added to PRP that was derived from the *Pitpα* ^*fl/fl*^ *Pf4-Cre* ^*+*^ mice, we observed 80% less thrombin generation when compared to PRP derived from the controls (Fig. 4d, e). This effect was platelet-dependent, since thrombin generation in platelet poor plasma (PPP) was identical between the two genotypes. We also analyzed the ability of *Pitpα* ^*fl/fl*^ *Pf4-Cre* ^*+*^ mouse-derived PRP to support thrombin generation when stimulated by standard tissue factors, and we found similar, albeit smaller, defects (Supplementary Fig. 6). A peculiar finding is that thrombin generation is further suppressed in PRP from *Pitpα* ^*fl/fl*^ *Pf4-Cre* ^*+*^ mice in comparison to that seen in PPP (Fig. 4d). Although the explanation for this observation is unclear, it suggests that platelets lacking PITPα suppress the ability of tumor cell tissue factor to initiate coagulation.

Under normal circumstances, generation of IP_3_ within platelets stimulates a rise in cytoplasmic calcium, which in turn exposes phosphatidylserine on the surface of platelets to support thrombin generation. We hypothesized that the defect in thrombin generation found in *Pitpα* ^*fl/fl*^ *Pf4-Cre* ^*+*^ plasma was due to impaired IP_3_ production, as shown previously, which caused low cytoplasmic calcium concentrations. To test this hypothesis, we artificially raised the intracellular calcium concentration and analyzed thrombin generation in PRP. We used ionophore A23187, which transports extracellular calcium into the platelet cytoplasm, as well as thapsigargin (TG), which can mobilize intracellular calcium by an IP_3_-independent mechanism^36, 37^. Both reagents have been demonstrated to activate platelets by increasing cytoplasmic free calcium levels^38–40^. Both *Pitpα* ^*fl/fl*^ *Pf4-Cre* ^*+*^ and *Pitpα* ^*fl/fl*^ *Pf4-* *Cre* ^−^ PRP generated a similar level of thrombin in the presence of the ionophore (10 µM) or TG (2 µM) (Fig. 4f, g). This demonstrates that increasing the intracellular calcium completely reverts the thrombin generation defect induced by the loss of PITPα. Thus, PITPα within platelets contributes to tumor metastasis through a signaling pathway that involves IP_3_ formation, calcium influx, phosphatidylserine externalization, and thrombin generation. Together, these data demonstrate that platelet PITPα is required for tumor-induced thrombin generation in vitro and thrombus formation in vivo.

### Platelet PITPα impairs the development of bronchus-associated lymphoid tissue (BALT) hyperplasia

**Fig. 5:**
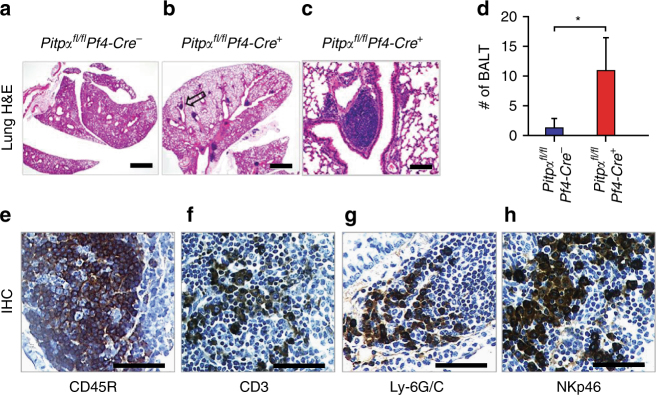
Loss of PITPα in platelets leads to a mucosal immune response. **a**–**c** Lung tissues were harvested 48 h after tumor injection and stained with H&E. *Pitpα* ^*fl/fl*^ *Pf4-Cre* ^*+*^ mice lacking PITPα in their platelets (**b**) demonstrate prominent formation of BALT (bronchus-associated lymphoid tissue or BALT hyperplasia), such as the one indicated by the arrow. This response was rarely observed in the control mice (**a**). A higher magnification of a typical BALT hyperplasia is shown in **c**. The black scale bars indicate 200 μm for **a**, **b**, and 50 μm for **c**. **d** Average number of BALT hyperplasia per section across 300 μm (*n* = 3 mice per group, *p < 0.05, unpaired Student’s *t*-test. Error bars are s.d.). **e**–**h** Immunohistochemistry staining of hyperplasia in *Pitpα* ^*fl/fl*^ *Pf4-Cre* ^*+*^ mice for major leukocyte makers as indicated. Black scale bars represent 200 µm

Although *Pitpα* ^*fl/fl*^ *Pf4-Cre* ^*+*^ mice developed less platelet–tumor adhesions than control mice during the first few hours after tumor injection (Fig. 3e–g), these results were not sufficient to explain the long-term effect of platelet PITPα on metastasis formation. To address this issue, we analyzed the histology of tumor metastasis over time. Two days after injection of the tumor cells, *Pitpα* ^*fl/fl*^ *Pf4-Cre* ^*+*^ mice developed strikingly hyperplastic BALT, a reaction that was essentially absent in the controls (Fig. 5a–d). This hyperplasia primarily consisted of hematopoietic-derived CD45R^+^ cells, including T cells (CD3^+^), neutrophils (Ly-6G/C^+^), and natural killer cells (NKp46^+^) (Fig. 5e–h). Thus, the initial stages of tumor implantation in mice lacking platelet PITPα are associated with inflammatory reactions that involve multiple types of leukocytes. These data suggest that signals generated by PITPα in platelets protect tumor cells from immune-mediated cytotoxicity, and thereby enable tumor cell survival and propagation in both the vasculature and in the tissues.

### Anticoagulation limits metastasis in *Pitpα*^*fl/fl*^*Pf4-Cre*^−^ mice

**Fig. 6:**
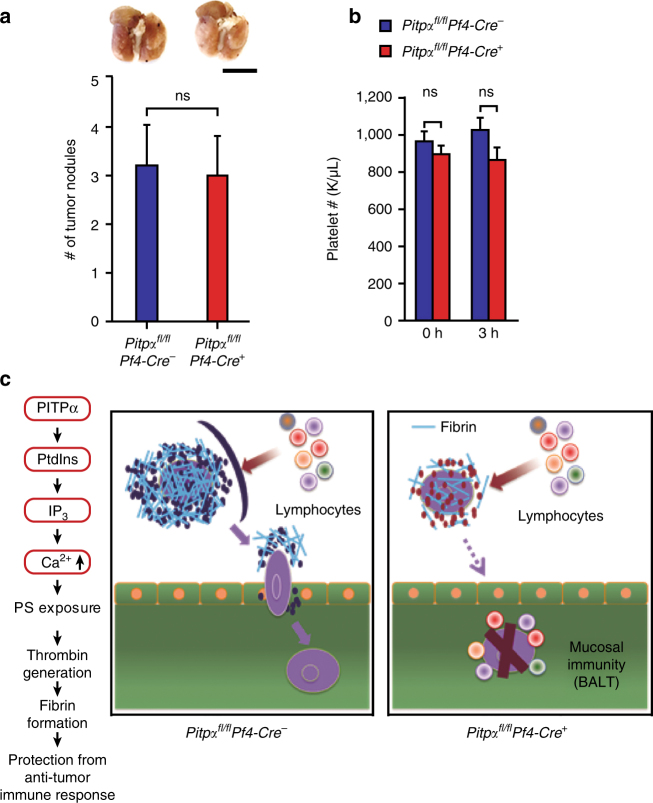
Coagulation inhibitor NAPc2 inhibits lung tumor metastasis in control mice. **a** Co-injection of tumors with coagulation factor VIIa inhibitor (NAPc2) reduced lung tumor metastasis in both groups checked 2 weeks after tumor injection (*n* = 10 per group). Scale bar = 1 mm. **b** Platelet counts at 3 h after injection of tumor cells together with coagulation factor VIIa inhibitor NAPc2 (*n* = 5 of each group). Shown are the means ± s.d. for **a**, **b**, and all statistical analysis was performed using an unpaired Student’s *t*-test. **c** A model of the sequential events that lead to protection of tumor cells from the immune system. Normal platelets rapidly adhere to the surface of tumor cells. This interaction facilitates prothrombinase assembly on the cell surface, thrombin formation, and the deposition of fibrin. This forms a shroud that protects the tumor from lymphocyte-mediated cytotoxicity. The platelets that coat the tumor cells also facilitate the adhesion and invasion involved in metastasis. However, platelets lacking PITPα adhere poorly to tumor cells, fail to stimulate thrombin generation, and reduce fibrin deposition. This allows the tumor cells to be vulnerable to lymphocyte-mediated cytotoxicity (such as BALT)

Tissue factor-initiated coagulation has been demonstrated to protect circulating tumor cells from elimination by immune surveillance, which facilitates tumor metastasis, and B16F10 melanoma cells express high levels of tissue factor^35^. To understand whether tumor cell-induced thrombin generation and fibrin formation mediated by PITPα in platelets contribute to metastasis, we investigated the effects of a coagulation FVIIa inhibitor, nematode anticoagulant protein c2 (NAPc2)^41^ injection on the lung metastasis formation of B16F10 melanoma cells in vivo. NAPc2 treatment in vivo inhibited lung metastasis when analyzed 2 weeks after injection in both genotypes. Anticoagulation reduced metastasis formation and thrombocytopenia in both genotypes (Fig. 6a, b). Furthermore, the effect of the PITPα mutation in platelets was completely reverted by anticoagulation. Histological analyses on lung tissue sections showed no significant thrombi formation at 3 h after injection, and no BALT hyperplasia developed 48 h after injection (Supplementary Fig. 7). Together, these data suggest that tumor-initiated coagulation contributes to tumor metastasis in normal mice, and that this effect can be impaired by either anticoagulation or by the PITPα mutation in platelets.

To confirm that the hemostatic process mediated by PITPα in platelets contributes to the lung metastasis of intravenous injected B16F10 melanoma cells, we investigated the impact of platelet PITPα on the growth of tumor cells injected subcutaneously into the flanks of mice. The data show that the flank tumors that developed in *Pitpα* ^*fl/fl*^ *Pf4-Cre* ^*+*^ mice are similar to those that developed in the control mice (Supplementary Fig. 8), demonstrating that tumor cells grow at equivalent rates within the two groups. Stated alternatively, since this model does not depend on coagulation or mucosal immunity, there is no influence on tumor formation by the loss of PITPα in platelets.

## Discussion

Clinical observations and experimental animal models have demonstrated that platelets support tumor metastasis formation. Our work confirms these findings and expands on these previous studies by establishing three additional and surprising observations: (1) Loss of PITPα in platelets causes only a partial deficit in phosphoinositide synthesis, yet it results in a major defect in second messenger formation; (2) In spite of a major biochemical defect in PITPα-null platelets, there is no obvious hemostatic defect; and (3) The loss of PITPα in platelets leads to a defect in tumor-induced thrombi formation that affects anti-tumor immunity. The data shown in this study demonstrate that PITPα-mediated phospholipid signaling in platelets contributes to lung metastasis of intravenous injected tumor cells, but has no significant impact on hemostasis. Together, this demonstrates that signaling pathways in platelets that are important for hemostasis can be distinct from those pathways involved in non-hemostatic functions.

Our data demonstrates that the loss of PITPα leads to the moderate loss of PtdIns(4,5)P_2_, but curiously leads to a far greater loss of its product, IP_3_. This suggests that PITPα is generating a particular pool of PtdIns(4,5)P_2_ that is critical for the formation of second messengers, such as IP_3_. Our previous work on PIP5KI (the enzyme required for the final step of PtdIns(4,5)P_2_ synthesis) demonstrated that second messengers, including IP_3_, are generated exclusively from a pool of recently synthesized PtdIns(4,5)P_2_^14^. Together, these data show that in order to make second messengers, platelets require a cooperative effect between PITPα and PIP5KI to rapidly synthesize PtdIns(4,5)P_2_ in discrete microdomains.

Since PITPα is required to generate normal amounts of phosphoinositides and second messengers within platelets, we anticipated finding defects in platelet-mediated hemostasis. However, we were surprised to see that the substantial amount of phosphoinositide synthesis mediated by PITPα does not contribute to pathways required for in vivo or ex vivo hemostasis. Our previous work has demonstrated that the loss of specific pools of PtdIns(4,5)P_2_ produced by PIP5KI within microdomains of platelets led to discrete platelet defects. Similarly, we speculate that microdomains of PtdIns(4,5)P_2_ production, that are regulated by PITPα, are tightly coupled to calcium levels in proximity to PS scramblases. In this scenario, the loss of PITPα would only diminish the pool of phosphoinositides required for PS exposure, and with the remaining pools of phosphoinositides, calcium and PS exposure would be sufficient for normal hemostatic function. Alternatively stated, we hypothesize that PITPα contributes to the synthesis of discrete pools of phosphoinositides within specific microdomains in platelets, and these pools are not required for hemostasis.

We observed that platelets lacking PITPα do not efficiently adhere to tumor cells in vivo and ex vivo by a mechanism that is related to a deficiency in thrombin generation and fibrin formation. As shown in model (Fig. 6c), PITPα-null platelets cannot form platelet–fibrin complexes that usually envelope tumor cells. This prevents thrombocytopenia in *Pitpα* ^*fl/fl*^ *Pf4-Cre* ^*+*^ mice following tumor cell injection. Without the platelet–fibrin shroud surrounding the tumors, these malignant cells are more vulnerable to mucosal immune responses that block tumor cell implantation. The evidence shown in this study demonstrates that impaired IP_3_-induced calcium release is the critical mechanism that prevents metastasis in *Pitpα* ^*fl/fl*^ *Pf4-Cre* ^*+*^ mice. Influx of intracellular calcium activates membrane scramblase activity and exposes phosphatidylserine on the platelet surface. This facilitates tenase and prothrombinase complexes that lead to thrombin generation and fibrin polymerization around the tumor cells. Together, these findings demonstrate that PITPα-mediated signaling in platelets indirectly augments the early stages of tumor dissemination.

While dysfunction of PITPα-null platelets seems to be the major contributor for decreased metastasis, it is important to note that there was a 15% decrease in platelet count for these mice. Both clinically and experimentally, this small degree of thrombocytopenia was not found to be critical for hemostasis. In addition, the data in this study demonstrate that the mild thrombocytopenia in *Pitpα* ^*fl/fl*^ *Pf4-Cre* ^*+*^ mice also did not contribute to the dissemination of tumors. This is based on the observation of the pronounced acute thrombocytopenia in control mice after tumor injection that was not evident in the *Pitpα* ^*fl/fl*^ *Pf4-Cre* ^*+*^ mice (Fig. 3d). Consequently, when compared to the control mice, there were actually more platelets in the circulation of the *Pitpα* ^*fl/fl*^ *Pf4-Cre* ^*+*^ mice during the critical early hours after tumor injection even though the metastasis defect was occurring. Thus, the inability of the tumors to metastasize could not be due to mild basal thrombocytopenia in the *Pitpα* ^*fl/fl*^ *Pf4-Cre* ^*+*^ mice, but rather due to an intrinsic defect of platelet function.

In 1865, Dr. Armand Trousseau first described the association between cancer and thrombosis^42^. Ironically, years later, Dr. Trousseau himself tragically succumbed to widespread thrombosis caused by his pancreatic cancer. Tumor cells are known to activate platelets, which in turn facilitates tumor implantation, adhesion, propagation, and invasion of the immune system. Our study demonstrates the vital role of platelet phosphoinositide signaling in the dissemination of cancer. Furthermore, this work also clearly distinguishes the platelet signaling processes required for hemostasis from those processes that augment metastasis formation. Additional studies will be required to determine whether targeting specific non-hemostatic platelet signaling pathways could be exploited in the development of clinical therapeutics.

## Methods

### Mice

Animals were maintained on standard chow and tap water. Animal procedures and experiments were approved by the Institutional Animal Care and Use Committee of the University of Pennsylvania. *Pitpα* conditional mice, *Flp* mice, and *Pf4-Cre* transgenic mice were used in this study. The genetic background of all mice used was C57BL6, and both sexes of mice were used for all experiments unless noted in the methods. The ages of mice used were between 8 and 20 weeks. To produce mice with conditional targeting of the murine *Pitpα* gene, a 10.8 kb genomic fragment was identified in a C57BL/6 (RPCI23: 105D24) BAC library and then cloned into the pSP72 vector (Promega). The “long homology arm” was 7.42 kb in length, and the “short homology arm” was 1.83 kb in length. The pGK-gb2 loxP/FRT Neo cassette was inserted into a region that corresponded to the 3′ end of exon 10, and an additional loxP site was inserted into a region that corresponded to the 5′ end of exon 8. Therefore, this targeting design removed a 1.55 kb region of *Pitpα* genomic DNA that included exons 8, 9, and 10. The targeting vector was confirmed by PCR, restriction analysis, and sequencing after each modification step. Additional confirmation of the final clones was performed by Southern blotting analysis.

The neomycin cassette flanked by FRT sites was removed by crossing with *flippase* (FLP) transgenic mice (Jackson Lab). Transgenic mice that contain Cre recombinase driven by platelet factor-4 promoter (*Pf4-Cre*) (obtained as a generous gift from Radek Skoda, of the University of Basel, Switzerland) were further crossed with *Pitpα* conditional mice to induce the deletion of PITPα, specifically in platelets and in megakaryocytes.

Genomic DNA isolated from tail biopsies was used for the genotyping of the conditional alleles of the *Pitpα* gene by PCR, using forward primer (5′-GAACAAGAAACTATCCAGCAGACAGACT-3′) and reverse primer (5′-CTTCCTCTGCCTTGTAATCCTGAG-3′).

### Antibodies and reagents

Antibodies used in this study were as follows: mouse anti-human PITPα (5F12) (Santa Cruz Biotechnology, sc-13569; 1:400); rabbit anti-human PITPβ (Abcam, ab83795; 1:1000); rabbit anti-β-Actin (Cell Signaling, #4970; 1:2500); goat anti-mouse TRP1 (A-20) (Santa Cruz Biotechnology, sc-10446; 1:150); rat anti-mouse CD41 (MWReg30) (Santa Cruz Biotechnology, sc-19963; 1:50); rat Anti-CD41 F(ab)2 fragments (MWReg30) (BD Biosciences, custom order; 0.12 µg/g body weight); anti-P-selectin (RB40.34) (BD Bioscience, custom order; 0.2 µg/g body weight); rat anti-mouse neutrophils (Ly-6G/C) (Santa Cruz Biotechnology, sc-59338; 1:50); rat anti-mouse CD45R (RA3-6B2) (Santa Cruz Biotechnology, sc-19597; 1:150); rat anti-mouse CD3 (17A2) (BioLegend, 100212; 1:150); rat anti-mouse CD335 (NKp46) (BioLegend, 137603; 1:120); anti-mouse fibrin (clone 59D8, gift from Dr. Rodney M. Camire of Children Hospital of Philadelphia, 1:500); PE-anti-mouse/rat CD62P P-selectin (RMP-1) (BioLegend, 148305; 1:150); PE-labeled rat anti-mouse integrin αIIbβ3 antibody (JON/A) (Emfret Analytics, #M023-2; 1:20); ImmPRESS reagent Anti-Rat Ig (MP-7404, Vector Laboratories); and ImmPRESS reagent anti-Goat Ig (MP-7405, Vector Laboratories).

Reagents used in this study were: Fura-2/AM (Thermo Fisher Scientific, F1221); Pluronic F-127 (Sigma, P2443); purified recombinant human PITPα and PITPβ proteins (a generous gift from Dr. Shamshad Cockcroft of University College London); CHRONO-LUME luciferase Luciferin (CHRONO-LOG, #393); Thrombin (CHRONO-LOG, #386); Collagen (Sigma, #885-1); Inositol-1,4,5-triphosphate [^3^H] radioreceptor assay kit (PerkinElmer Life Science, #NEK064); and Phosphorus-32 Radionuclide, 2 mCi (PerkinElmer Life Science, #NEX053H002MC).

### Washed platelet preparation and aggregation

Washed platelets were prepared from blood collected from the inferior vena cava of anesthetized mice in the presence of 6× acid citrate dextrose buffer (ACD, pH 4.4, 85 mM sodium citrate dihydrate, 66.6 mM citric acid, 111 mM dextrose)^14^. All procedures were done at room temperature. PRP was separated by centrifugation of blood at 200 × *g* for 10 min. PRP was incubated with 1 μM prostaglandin E1 (PGE1) for 10 min. Platelets were isolated by centrifugation of PRP at 800 × *g* for 10 min to remove PPP. The pellet was resuspended in Hepes-Tyrode buffer pH 7.4 (134 mM NaCl, 3 mM KCl, 0.3 mM NaH_2_PO_4_, 12 mM NaHCO_3_, 2 mM MgCl_2_, 5 mM Hepes, 5 mM glucose, and 0.35% BSA). For the aggregation assay, washed platelets were adjusted to 2 × 10^8^ platelets/ml by using Hepes-Tyrode’s buffer and supplemented with 1 mM CaCl_2_. The aggregation was measured by the turbidometric method at 37 °C in the presence of CHRONO-LUME luciferase in a Lumi-Dual aggregometer (Chrono-Log).

### Quantitation of platelet phosphoinositides

Platelets were prepared as described above and were washed once with modified Tyrode’s buffer (pH 6.5) (134 mM NaCl, 3 mM KCl, 0.3 mM NaH_2_PO_4_, 12 mM NaHCO_3_, 2 mM MgCl_2_, 1 mM EGTA, 1 μM PGE1, 5 mM Hepes, 5 mM glucose, and 0.35% BSA). The platelets were then resuspended to 1 × 10^9^/ml by using modified Tyrode’s buffer (pH 7.4) without PGE1, EGTA, and NaH_2_PO_4_. The platelets were supplemented with 1 mM CaCl_2_. Platelet suspensions (0.5 ml) were incubated (phosphate-starved) at 37 °C for 30 min and then supplemented with 0.5 mCi of ^32^P for 90 min. The incorporation of ^32^P was halted by the addition of 50 μl of ice-cold HCl and 1 volume of methanol:chloroform (1:1) on ice. Mixtures were vortexed for 1 min to extract total phospholipids and were centrifuged at 2000 × *g* for 3 min. The chloroform phase was isolated, and the remaining aqueous phase was extracted again with chloroform. The two chloroform phases were combined and evaporated under N_2_ flow. The dried residue was resuspended in 25 μl of ice-cold chloroform:methanol (1:1). Total phospholipids in the chloroform/methanol solution were fractionated by TLC and analyzed as described^14^. The levels of each phosphoinositide were quantified by JustTLC software (Sweday).

### Quantitation of IP_3_ formation in platelets

The washed platelets were prepared as described above, and their density was adjusted to 7–10 × 10^8^/ml in Hepes-Tyrode’s buffer that was supplemented with 1 mM CaCl_2_. Platelets in 200 μl aliquots were activated by thrombin at 1 U/ml for indicated times. The reaction was halted by adding 40 μl of ice-cold 20% perchloric acid, and incubated for 20 min to lyse the cells. The cellular debris was removed by centrifugation at 2000 × *g* for 15 min, and the supernatant was adjusted to pH 7.5 by the addition of KOH, and then clarified again at 2000 × *g* for 15 min at 4 °C before harvesting the final supernatant. Quantitation of IP_3_ was performed by using the inositol-1,4,5-trisphosphate [^3^H] radio-receptor assay kit (PerkinElmer), according to the manufacturer’s protocol.

### In vivo thrombosis tail bleeding time

The distal 5 mm tail segment of 5–6-weeks-old mice were amputated with a scalpel after administration of anesthesia. The tails were immediately immersed in 37 °C saline. The bleeding time was presented as the sum of bleeding within 15 min of observation, including re-bleeding. The time of complete cessation of blood flow (i.e., no blood flow for 1 min) was noted. The lost blood was collected in PBS and the blood cells were lysed in Red Blood Cell Lysing buffer (Sigma-Aldrich, R7757) to collect protein lysate. The blood volume was represented by the measurement of hemoglobin in the lysate of lost red blood cells^43^.

### FeCl_3_-induced carotid thrombosis formation

Mice were anesthetized using 2% isoflurane gas in oxygen. A midline incision was made in the neck, and the right carotid artery was exposed by blunt dissection. Whatman filter paper (#1, 1 mm^2^) soaked in 7.5% FeCl_3_ was applied to the exposed artery for 2 min. After removal of the filter paper, the artery was washed with PBS, and blood flow was recorded using a small animal blood flow meter (model T106, Transonic Systems Inc., Ithaca, NY) for 30 min. The time to full occlusion is reported.

### Laser-induced in vivo thrombosis

Male mice 8–12 weeks of age were anesthetized via intraperitoneal injection of sodium pentobarbital (90 mg/kg)^30^. A cannula was introduced into the jugular vein for the delivery of antibodies and additional anesthetic as needed. The cremaster muscle was exteriorized, cleaned of connective tissue, opened, and spread flat on the glass coverslip of a custom-built chamber for viewing by intravital microscopy. The cremaster preparation was continuously superfused with bicarbonate buffer warmed to 36.5 °C and bubbled with 95% N_2_/5% CO_2_. The cremaster microcirculation was visualized using an Olympus BX61WI upright microscope with a 60× (0.9 NA) water immersion objective, coupled to a Yokogawa CSU-X1 spinning disk confocal scanner. Laser injury was induced with a pulsed nitrogen dye laser (SRS NL100, 440 nm, 55-65% power setting) focused on the vessel wall by the microscope objective. The laser fired at the vessel wall until only a small number of red blood cells exited the lumen of the vessel (1–10 laser pulses). Confocal fluorescence images were acquired using a CoolSnap HQ CCD digital camera (Photometrics, Tucson, AZ). The microscope, confocal scanner, lasers, and camera were all controlled and synchronized using Slidebook 5.0 image acquisition and analysis software (Intelligent Imaging Innovations). Arterioles (30–40 µm diameter) with unperturbed blood flow were selected for study. Anti-CD41 F(ab)2 fragments (0.12 µg/g body weight; clone MWReg30, BD Bioscience) and anti-P-selectin (0.2 µg/g body weight; clone RB40.34, BD Bioscience) antibodies were labeled with Alexa fluor dye monoclonal antibody labeling kits (Alexa-488, Alexa-568, and Alexa-647), according to the manufacturer’s instructions (Invitrogen).

### Quantitative analysis of the lung metastasis of B16F10 melanomas

The B16F10 cell line was obtained from the American Type Culture Collection (Manassas, VA, USA) and cultured in Dulbecco’s Modified Eagle’s Medium that was supplemented with 10% v/v, fetal calf serum, and 300 μg/ml L-glutamine. Tumor cells from a mid-log phase culture were collected by brief exposure to 0.05% trypsin/EDTA solution (Invitrogen), washed twice with PBS, and then re-suspended in PBS at a density of 5 × 10^6^/ml. A 200 μl aliquot of B16F10 tumor cells (1 × 10^5^ cells) with or without FVIIa inhibitor NAPc2 (150 nM) was injected through the tail veins of the mice. At various time intervals after injection, the mice were euthanized, and perfused with 10 ml ice-cold PBS that was supplemented with 4% formalin. The lungs were carefully removed, dissected free of other connective tissues, and then fixed in 10% formalin. The tumor nodules on the lung surface were counted under microscopy, and the lung was weighed.

### Quantification of thrombus formation

Tumor injection-induced thrombi formations in lung tissue were quantified on paraffin-embedded tissue sections. Three slides across 300 μm were collected in each sample at 100 μm intervals. Platelet-rich thrombi were identified by immunohistochemistry staining of CD41, and the numbers either in the vasculature or within the pulmonary tissue were counted under the microscope at 10× magnification from three randomly selected optical fields.

### Immunohistochemistry

The slides were de-paraffinized with xylene, dehydrated in ethanol, and then treated with 20 μg/ml proteinase K in TE buffer, pH 8.0 containing 0.5% triton X-100 for 15 min at 37 °C. The slides were then treated with 3% H_2_O_2_ for 15 min to block endogenous peroxidase, and then treated with 2.5% normal serum for 60 min to block non-specific antibody binding. The tissue sections were stained with primary antibodies and peroxidase secondary antibody as instructed by the ImmPRESS reagent kit (Vector Laboratories). The signals were visualized by developing with DAB as indicated by the ImmPact DAB peroxidase substrate kit (Vector Laboratories).

### Immunoblotting

Washed platelets were prepared as described above. After final centrifugation, the platelet pellet was resuspended in ice-cold RIPA buffer (Sigma-Aldrich, R0278) supplemented with cOmplete protease inhibitor (Roche, 11836170001) to lyse the platelets. Platelet lysate protein concentration was measured with BCA kit (Thermo Scientific Pierce, 23225) and adjusted with RIPA buffer so that each sample had a total of 50 µg of protein. NuPage LDS Sample buffer (Invitrogen, NP0008) and NuPage reducing agent (Invitrogen, NP0004) were added to each sample aliquot. Samples were loaded on a NuPage 4–12% Bis-Tris protein gel (Invitrogen, NP0321) and run in reducing conditions with a NuPage MOPS SDS running buffer (Invitrogen, NP0001). Gels were then blotted onto polyvinylidene fluoride membrane (Invitrogen, LC2002). After blotting, membranes were blocked with 5% Blotting Grade Blocker Non-Fat Dry Milk (BioRad #1706404XTU) in 0.1% Tween/TBS (BioRad #1706435, #1662404) for 1 h. Blots were incubated with primary antibody in blocking buffer overnight at 4 °C. Afterwards, blots were incubated with secondary antibody conjugated with HRP (anti-rabbit or anti-mouse; Cell Signaling Technology, #7074, #7076) for 1 h at room temperature. Blots were washed several times with 0.1% Tween/TBS between each step. Blots were developed with ECL Prime Western Blotting detection reagent (GE Healthcare Life Sciences, RPN2232), exposed on autoradiographic film (Denville Scientific, E3018), and digitized on a scanner. Uncropped immunoblots are shown in Supplementary Fig. 9.

### Annexin V binding on agonist-activated platelets

The binding of Annexin V on platelets was quantified by flow cytometry^44^. Whole blood was collected from the inferior vena cava and diluted at 1:100 in the final resuspension buffer (pH 7.4, 134 mM NaCl, 3 mM KCl, 0.3 mM NaH_2_PO_4_, 12 mM NaHCO_3_, 2 mM MgCl_2_, 5 mM Hepes, 5 mM glucose, and 0.35% BSA) that contained 2 mM CaCl_2_. GPRP (Sigma, Cat# G1895-25mg) at a final concentration of 1 mM was supplemented to prevent fibrin polymerization before the stimulation of agonists. Diluted platelets in whole blood were stimulated with an agonist combination that contained 5 μg/ml collagen and 0.1 U/ml thrombin at 37 °C for 10 min. The samples were then labeled with 50 nM Annexin V-Alexa-FITC antibody (BD Biosciences, #556420; 1:20) and CD41a-PE antibody (BD Biosciences, #558040; 1:100) to identify platelets at room temperature for 30 min. The binding was analyzed by flow cytometry (BD FACSCanto II Flow Cytometry System) gating on the CD41-positive cell population.

### Flow cytometry

After collecting platelets, as described above, cells were diluted to 10^7^ cells/ml. Platelets (100 µl) were stained with PE-labeled rat anti-mouse integrin αIIbβ3 antibody (JON/A) (Emfret Analytics, #M023-2; 1:20) for 30 min. The tubes were washed with 0.1% BSA in PBS and centrifuged to remove non-bound antibody and resuspended in 0.1% BSA in PBS before analyzing cells on FACSCanto II Flow Cytometry System (BD Bioscience). Flow cytometry was assisted by the Flow Cytometry Core Laboratory at Children’s Hospital of Philadelphia core facility.

### Thrombin generation assay

Thrombin generation in platelets was measured in the automated analyzer CEVERON alpha with a TGA module (TECHNOTHROMBIN TGA, DiaPharma) based on monitoring the formation of thrombin by means of a fluorogenic substrate upon activation of the coagulation cascade by tissue factor. Blood was withdrawn in the presence of 6× ACD, and PRP was collected after centrifuging at 200 × *g* for 10 min. The samples were further centrifuged for 15 min at 1500 × *g* to collect the PPP as controls. Platelets in PRP were counted, and the concentration was adjusted to 5 × 10^8^/ml. For each measurement, 40 μl of each sample, 10 μl of TGA trigger B16F10 tumor cells (1 × 10^7^/ml), and agonists were mixed in 96-well plates in duplicate. The samples were maintained at 33 °C. Once 50 μl of TGA substrate was added into each well, measurements were obtained at 30 s intervals for 90 min. Thrombin generation was calculated by the manufacturer’s software utilizing a standard thrombin calibration curve that was derived separately.

### Quantification of BALT hyperplasia

The BALT hyperplasia on each section was manually counted under microscope at 4× magnification after H&E staining. One section per 100 μm tissue was used for the quantification, and a total of three slides across 300 μm were collected.

### Platelet counts

Peripheral blood was collected through retro-orbital puncture of *Pitpα* ^*fl/fl*^ *Pf4-Cre* ^*+*^ and *Pitpα* ^*fl/fl*^ *Pf4-* *Cre* ^−^ mice matched for age and gender. Complete blood counts (CBC) and mean platelet volumes were analyzed using a Drew Hemavet Hemacytometer (HV1700).

### Intracellular calcium flux measurement

PRP was collected and platelets were suspended to 5 × 10^8^/ml in Tyrode’s buffer without calcium. Apyrase and PGE-1 were added to prevent aggregation. Platelets were loaded with fura-2/AM (5 μM) in the presence of Pluronic F-127 (0.2 μg/ml) for 15 min at 37 °C, then washed and resuspended in Tyrode’s buffer without calcium. Stirred platelets were activated with thrombin, and fluorescence was measured with an Aminco Bowman Series 2 Luminescence spectrophotometer^45^. The excitation wavelengths alternated between 340 and 380 nm, and emission was measured at 510 nm. Each measurement was calibrated using Triton X-100 and EGTA. Fura-2 *K*_d_ = 224 nM. Maximal increase in [Ca^2+^] was determined by subtracting baseline before stimulus from peak.

### Fibrin staining

Fibrin production in thrombi was stained by rapid microwave phosphotungstic acid hematoxylin (P.T.A.H) (American MasterTech) on 6 μm paraffin-embedded sections of lung tissue. De-paraffinized slides were incubated in microwave heated 10% zinc chloride solution for 15 min, washed in running tap water, and followed by incubation in microwave heated 5% ferric ammonium sulfate solution for 2 min. After washing with water, the slides were further stained for 30 min in P.T.A.H that had been pre-heated in a microwave for 10–20 s. The slides were dehydrated in 95% alcohol and then covered with a mounting slip prior to microscope analysis.

### Lung metastatic analysis of intravenous injected LLC

LLC cell line was obtained from the American Type Culture Collection (Manassas, VA, USA, #CRL-1642) and cultured in Dulbecco’s Modified Eagle’s Medium that was supplemented with 10% v/v, fetal calf serum (Sigma Aldrich), and 300 μg/ml L-glutamine (Invitrogen). Tumor cells from a mid-log phase culture were collected by brief exposure to 0.05% trypsin/EDTA solution, washed twice with PBS, and then re-suspended in PBS at a density of 5 × 10^6^/ml. A 200 μl aliquot of tumor cells (1 × 10^5^ cells) was injected through the tail vein of the mice. The mice were euthanized at 3 weeks after injection, and perfused with 10 ml ice-cold PBS that was supplemented with 4% formalin. The lungs were carefully removed, dissected free of other connective tissues, and then fixed in 10% formalin. The tumor nodules on the lung surface were counted under microscopy. Some of the lungs were paraffin-embedded and sectioned at 6 µm for H&E staining. The tumors on tissue sections were counted under microscope at 10× magnification across 300 µm of each lung and presented as average number of tumors per section.

### Flank tumors

B16F10 melanoma cells were utilized for flank tumor growth measurement. Tumor cells at 1 × 10^6^/ml in PBS were subcutaneously injected. Two weeks after injection, mice were euthanized and the tumors were dissected, weighed, and fixed in 10% formalin.

### Statistics

Two-tailed unpaired or paired Student’s *t*-tests were applied for the comparison of two means for all analyses, except for the tail bleeding times and intravital assay, which utilized the Mann–Whitney test. *P-*values of less than 0.05 were considered statistically significant.

### Data availability

All relevant data are available from the authors upon reasonable request.

## Electronic supplementary material

Supplementary Information
