# Supplementary material for: Phosphatidylinositol transfer protein-α in platelets is inconsequential for thrombosis yet is utilized for tumor metastasis
Source: Nat Commun. 2017 Oct 31;8:1216. doi: 10.1038/s41467-017-01181-4 (PMC5662573; doi:10.1038/s41467-017-01181-4)
Supplement: Supplementary file 1 — Supplementary Information [file 41467_2017_1181_MOESM1_ESM.pdf]

SUPPLEMENTARY FIGURES

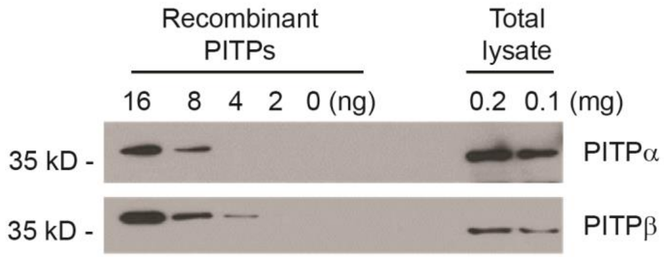

Supplementary Figure 1. Immunoblots demonstrating PITPα and PITPβ expression in murine platelet lysates using isoform specific antibodies. Shown is the mass of the recombinant protein, and the total mass of platelet lysates loaded in each lane.

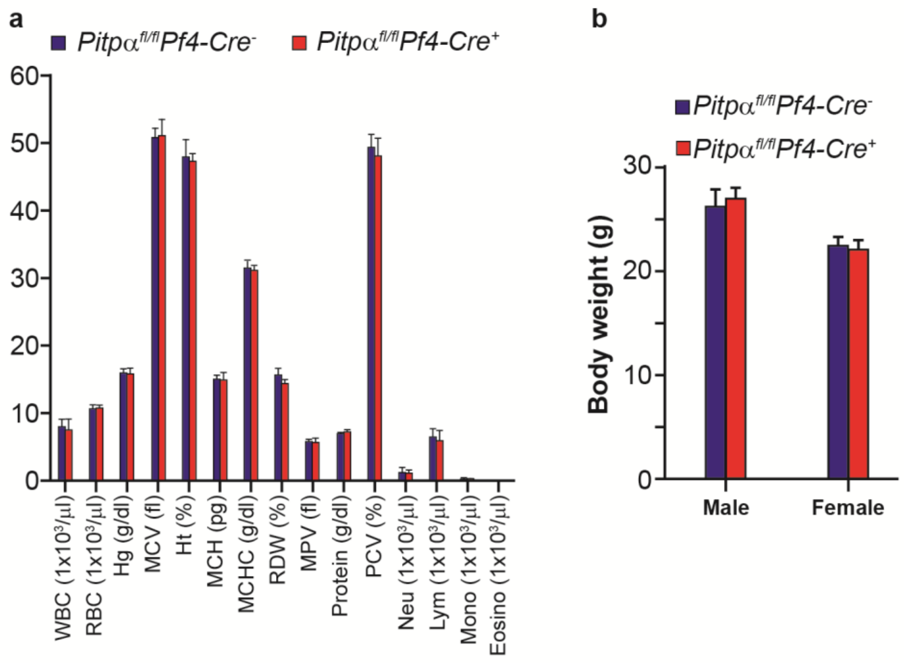

Supplementary Figure 2. (**a**) CBC and (**b**) body weight analysis of *Pitpα*$^{fl/fl}$*Pf4-Cre*$^{+}$ mice compared to their littermate controls. Error bars are s.d.

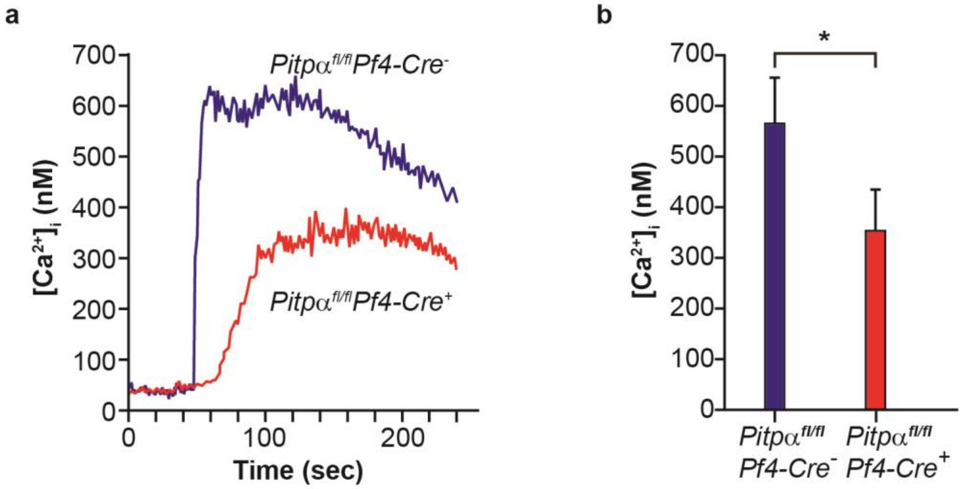

Supplementary Figure 3. Intracellular calcium measurements in platelets. (**a**) Representative trace of fura-2-loaded *Pitpα*$^{fl/fl}$*Pf4-Cre*$^{-}$ (blue) and *Pitpα*$^{fl/fl}$*Pf4-Cre*$^{+}$ (red) platelets stimulated with 1 U/ml thrombin at 55 seconds and monitored by luminescence spectrophotometer. (**b**) Maximal increase in $[Ca^{2+}]_i$ ± SD when stimulated with 1 U/ml thrombin (* $p < 0.05$, unpaired Student's t-test, $n = 3$ mice per group). Error bars are s.d.

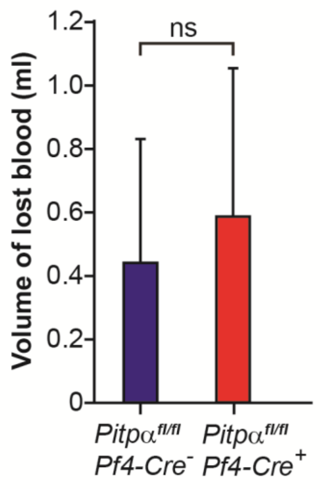

Supplementary Figure 4. The volume of lost blood in tail bleeding assay is represented by the measurement of hemoglobin in the lysates of lost red blood cells *Pitpα*$^{fl/fl}$*Pf4-Cre*$^{+}$ mice and in *Pitpα*$^{fl/fl}$*Pf4-Cre*$^{-}$ control mice (n = 3 mice per group, error bars are s.d., unpaired Student's t-test).

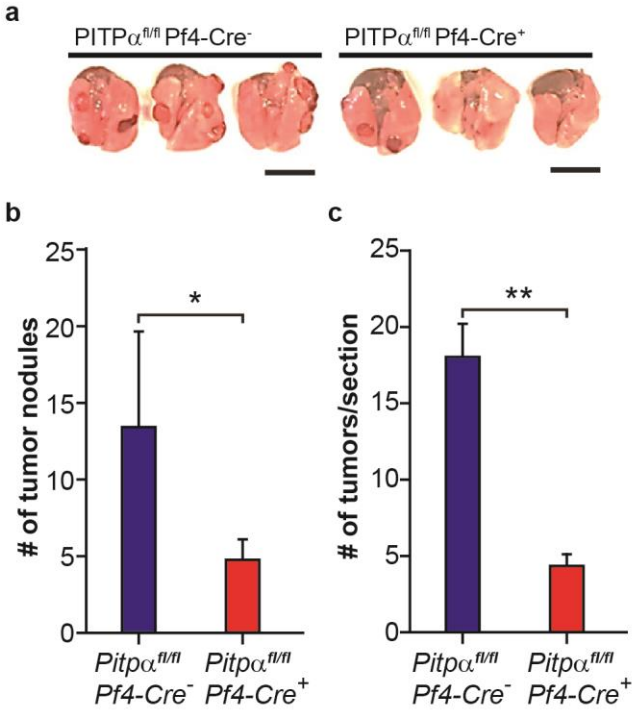

Supplementary Figure 5. Lung metastasis resulting from LLC cells injected intravenously into mice lacking PITPα in platelets. (**a**) Representative lung metastasis of LLC three weeks after tumor injection. (**b**) Quantification of tumor nodules on the surface of lung tissue ($n = 10$ for each group). (**c**) Quantification of tumors in lung tissue sections. Each data point is the average tumor number per section across a total of 200 microns ($n = 9$ for each group) at two weeks after injection. (**b, c**) p * < 0.01, p ** < 0.0005, unpaired Student's t-test. Error bars are s.d.

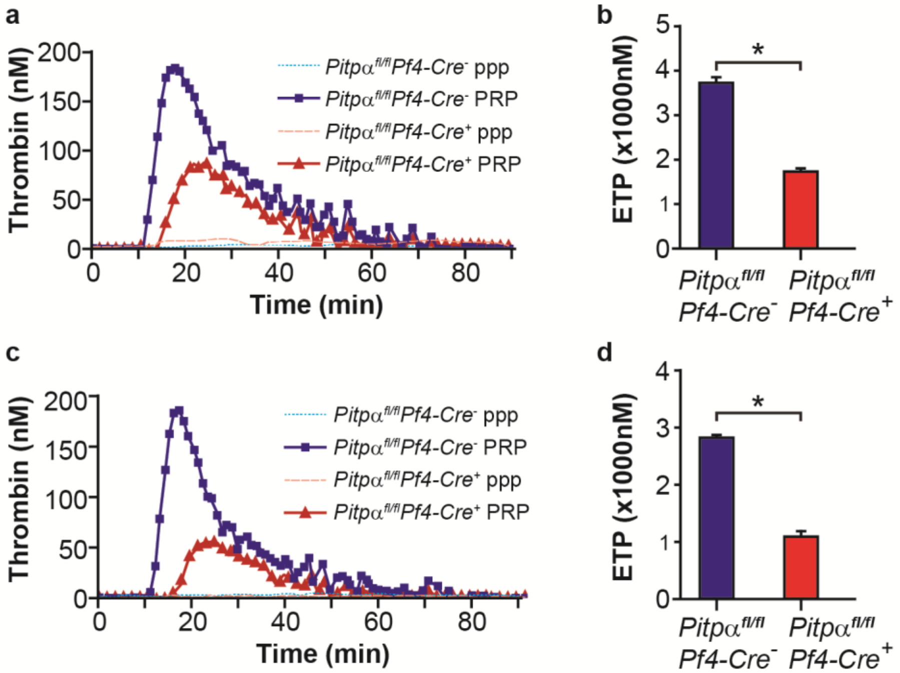

Supplementary Figure 6. Loss of PITPα impairs *ex vivo* thrombin generation. TGA initiated by the Diapharma reagents TF/RB (**a**, **b**) and TF/RC (**c**, **d**). Shown are representative kinetics of thrombin generation in PRP and in PPP (**a**, **c**). Note that PPP did not generate any thrombin in response to either thrombin generation reagents. The ETP shown in **b** and **d** is the mean value ± s.d. of total thrombin generated in PRP, which contains either PITPα control platelets (blue bar) or PITPα-null platelets (red bar). * $p < 0.01$. Statistical analysis was performed using an unpaired Student's t-test.

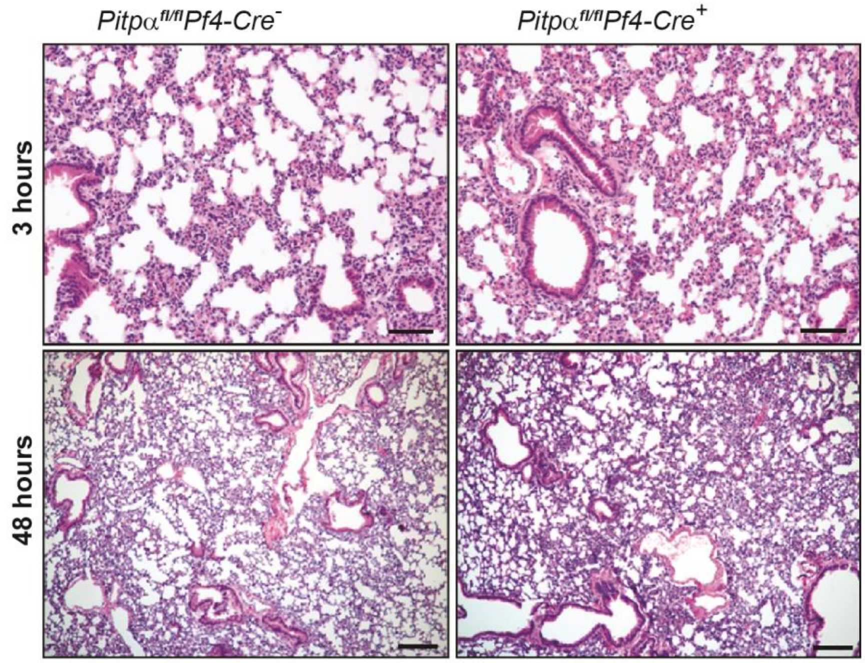

Supplementary Figure 7. H&E staining analysis on the impact of factor VIIa inhibitor NAPc2 on tumor-induced thrombi formation at 3 hours (10x) and BALT hyperplasia formation at 48 hours (4x) after tumor injection in *Pitpα$^{fl/fl}$Pf4-Cre^{+}* mice. Black scale bar represents 100 µm at 10X and 250 mm at 4X.

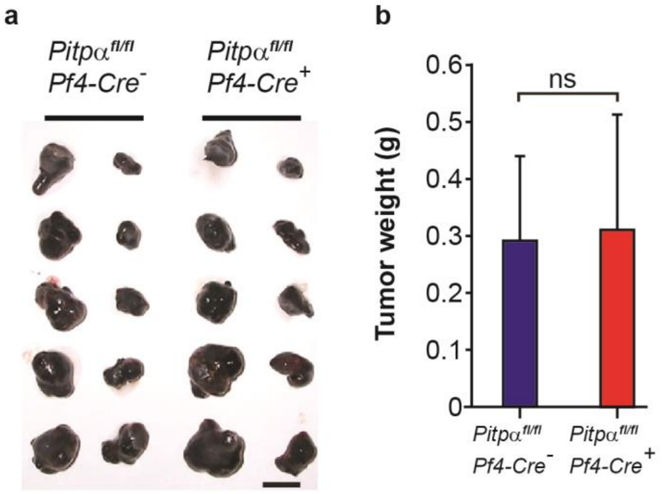

Supplementary Figure 8. (**a**) Flank tumors of subcutaneously injected B16F10 melanoma cells in *Pitpα*$^{fl/fl}$*Pf4-Cre*$^{+}$ mice and their controls. Scale bar = 1 mm. (**b**) The growth of tumors was represented by their weights. *N* = 3 per group. Shown are the mean tumor weights ± s.d. Statistical analysis was performed using an unpaired Student's t-test.

Fig. 1c

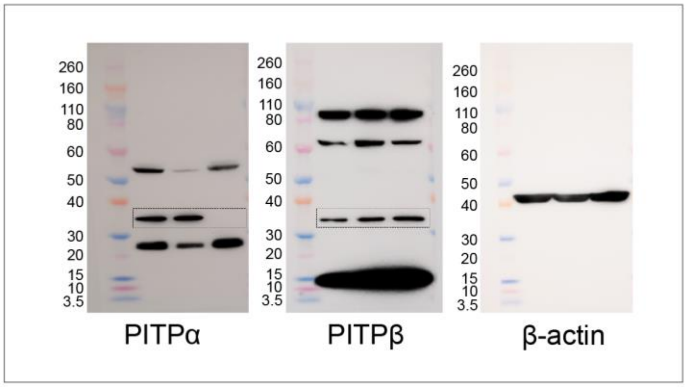

Fig. S1

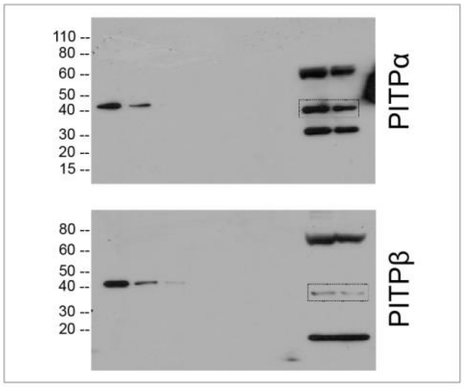

Supplementary Figure 9. Uncropped images of immunoblots used in this study.

Supplementary Table 1. Average peak *ex vivo* platelet aggregation values as measured with light transmission aggregometry after stimulation with agonists.

Agonists	Thrombin		Collagen		ADP		U46619		PMA	
	0.1u/mL	1u/mL	5µg/mL	10µg/mL	5µM	10µM	1.25µM	2.5µM	150nM	300nM
Pitpα$^{fl/fl}$ *Pf4-Cre*$^{-}$ (%)	61.5±3.00	78.6±3.21	52.3±7.37	65.3±1.52	19.3±3.21	36.6±1.53	46.5±3.10	73.0±3.60	60.5±5.26	71.7±6.23
Pitpα$^{fl/fl}$ *Pf4-Cre*$^{+}$ (%)	58.6±7.23	79.0±2.64	51.6±4.72	63.6±6.65	18.2±3.51	30.0±5.56	45.0±3.91	74.6±9.76	52.5±4.76	64.0±4.50
p-value	0.472	0.896	0.901	0.445	0.734	0.116	0.57	0.874	0.123	0.144

Supplementary Table 2. *Ex vivo* platelet integrin activation and secretion after stimulation. Surface integrin αIIbβ3 activation was measured by Jon/A antibody and flow cytometry. Alpha granule secretion was measured by P-selectin exposure with P-selectin antibody and flow cytometry. Shown are fold changes in relative fluorescent units.

	**JON-A**			**P-Selectin**		
	Pitpα $^{fl/fl}$ *Pf4-Cre* $^{-}$	*Pitpα* $^{fl/fl}$ *Pf4-Cre* $^{+}$	*p*-value	*Pitpα* $^{fl/fl}$ *Pf4-Cre* $^{-}$	*Pitpα* $^{fl/fl}$ *Pf4-Cre* $^{+}$	*p*-value
0.5uM ADP	2.018±0.937	2.103±1.090	0.8836	1.561±0.518	1.464±0.432	0.7549
5uM ADP	2.723±0.334	2.710±0.882	0.9696	2.119±0.813	1.674±0.692	0.3312
0.01U/mL Thrombin	1.246±0.072	1.200±0.125	0.5474	2.257±0.592	2.746±0.635	0.3841
0.1U/mL Thrombin	3.093±1.471	3.734±1.367	0.3976	13.203±9.302	15.589±12.624	0.7425
1U/mL Thrombin	4.147±1.383	4.445±2.793	0.8027	14.212±9.421	16.891±12.744	0.7152
